# The Italian value chain in the pandemic: the input–output impact of Covid-19 lockdown

**DOI:** 10.1007/s40812-020-00164-9

**Published:** 2020-07-06

**Authors:** Raffaele Giammetti, Luca Papi, Désirée Teobaldelli, Davide Ticchi

**Affiliations:** 1 Università Politecnica Delle Marche, Ancona, Italy; 2 Università Di Urbino, Urbino, Italy

**Keywords:** Covid-19, Lockdown, Global value chains, Production networks, C67, R15, F13, F14, O21

## Abstract

This paper investigates the role of the domestic value chain in transmitting the economic impact of Covid-19 lockdown measures. By employing techniques of complex networks analysis and input–output traditional tools, the study identifies those sectors that are key in the complex structure of the Italian supply chain and provides different rankings of the most ‘systemically important’ industries involved in the Covid-19 lockdown. The results suggest that by stopping the production process of many key sectors, the lockdown has led to a drop in input and output that, in turn, has generated a lock of about 52% of total circulating value added, 30% of which has been locked within indirect value chains. Further, by adding sectoral physical proximity indexes to the scenarios analysis, the method developed here provides a tool to guide governments in designing safe and efficient reopening policies.

**Electronic supplementary material:**

The online version of this article (10.1007/s40812-020-00164-9) contains supplementary material, which is available to authorized users.

## Introduction

The outbreak of the global Covid-19 pandemic has led many countries to implement drastic social distancing rules and sectoral lockdowns. While there is already a large strand of the literature that analyzed and provided evidence on the effectiveness of social distancing interventions in delaying and reducing the spread of epidemics (see, among others, Hatchett et al. 2007; Markel et al. 2007; Bootsma and Ferguson 2007), our knowledge of the economic impact of such public health measures is instead rather limited (e.g., Wren-Lewis 2020). However, one should also recognize that there has been a large number of works in the last few months that have tried to fill this gap along many dimensions. For example, some works have analyzed the trade-off between public health and output, others have studied the effects of pandemic on sectors most affected by government restrictions and/or by the economic fallout (e.g., tourism, banks, etc.), while a number of works have focused on the optimal fiscal and monetary policies aim at reducing the output and welfare losses (see Caracciolo et al. 2020, for a review).

In this paper we want to contribute to the strand of this fast-growing literature that studies the output losses generated by governments’ restrictions on economic activity. In doing this, our starting point is that any analyzes aiming at quantifying the economic impact of restrictions to production needs to take into account the interlinkages between sectors, a feature that is often mentioned in public debates but seldomly explicitly considered in economic analyses. Indeed, as Caracciolo et al. (2020, p. 10) conclude in their review: “The relevance of complementarities between sectors (e.g. input–output chains, or more simply those between the education system and parents’ capability of employability) is sometimes mentioned, but rarely taken into account.”

In particular, we here investigate the main features of the Italian production network and then quantify the role of the domestic value chain in the transmission of the economic impact of Covid-19 lockdown measures. As anticipated above, the stop to many production processes caused by lockdown measures leads to a drop in input and output whose economic impact are difficult to quantify without considering the interlinkages between sectors. For this reason, we employ the techniques of complex networks analysis and input–output traditional tools that allows us to identify the sectors that are key in the complex structure of the Italian supply chain and to provide different rankings of the most ‘systemically important’ industries involved in the Covid-19 lockdown measures.

The results of our analysis suggest that by stopping the production process of many key sectors, the lockdown has led to a drop in input and output that, in turn, has generated a lock of about 52% of total circulating value added, 30% of which has been locked within indirect value chains.^1^ Further, by adding sectoral physical proximity indexes to the scenarios analysis, the method developed here provides a tool to guide governments in designing safe and efficient reopening policies.

Our work is closely related to various recent works that have analyzed the impact of the Covid-19 pandemic on GDP. For example, the study of the OECD (2020), that aggregates industry-level shocks, estimates a potential immediate impact of shutdowns measures on GDP of around 25%. Barrot et al. (2020) estimate industry-level shocks by considering the list of essential industries in a multisector input–output framework, and find that 6 weeks of social distancing would bring GDP down by 5.6%. Similarly, Baqaee and Farhi (2020a, b) study the effects of the lockdown on GDP declines in input network economies and show how nonlinearities associated with complementarities in consumption and production amplify the effect of negative supply and demand shocks. Bonadio et al. (2020) analyze the role played by global supply chains in estimating the impact of the Covid-19 pandemic on GDP growth for 64 countries finding that cross-country variations are well-explained by differences in lockdown severity across countries. Our work differs from the above cited contributions along two dimensions. First, we focus on the Italian economy. And, second, while all these studies are based on assumptions about social distancing rules and lockdowns severity, we draw directly on the list of essential industries developed by the Italian Prime Minister’s Decree (IPMD) dated April 10, 2020.

The work is organized as follows. Section 2 explores and discusses the main features of the Italian production network. Section 3 describes the model and methodology used to assess the amount of GDP locked and discusses the results, while Sect. 4 offers some concluding remarks.

## A descriptive analysis of the Italian production network

The analysis of the domestic and global production chains is crucial in establishing whether and how the effect of sectoral lockdown can propagate throughout the economy and lead to significant aggregate fluctuations. In this section we summarize the main features of the Italian production network (IPN) and analyze the most central sectors.^2^

In order to build the IPN, we employ the symmetric input–output table (SIOT) provided by the Italian National Institute of Statistics (ISTAT) whose last released version refers to the year 2016 and includes 63 sectors classified by the Nace Rev. 2. As shown in Giammetti et al. (2020), Input–output tables provide a natural source of information for representing the economy as a network. In particular, we consider here the $63\times 63$ inter-industry transactions matrix as a weighted adjacency matrix of a network where the nodes are individual sectors and edges are euro goods flows within and across sectors. The direction of the flows goes from the supplier sector to the buyer sector.

**Fig. 1:**
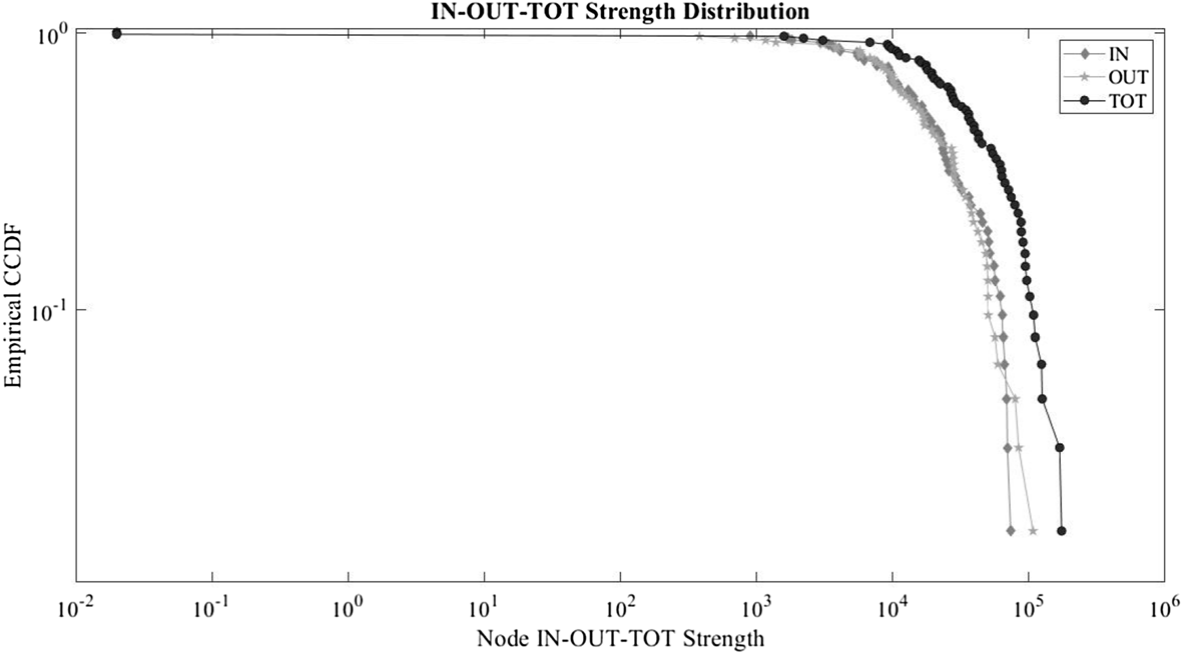
Node in–out and total strength distributions

Similarly, to multi-country production networks (Cerina et al. 2015; Giammetti et al. 2020), the IPN is featured by highly left-skewed degree distributions, showing that most of the sectors in the economy have many connections with other sectors. The average degree is about 60.48, i.e. every node is linked with almost every node. However, if we consider the weight of these connections and focus on the strength distributions things are different. Figure 1 illustrates the empirical distributions of in–out and total strength in the IPN. The *x*-axis is the in–out and total strength for each sector presented on a log scale. The *y*-axis, also in log scale, represents the probability that the sector *i*th has a strength larger than or equal to *x*. Hence, the upper left-hand portion of the graph shows that nearly 100% of sectors have an in–out and total strength greater than 0.01; moving down on the *y*-axis we see that only about one tenth of all sectors have an in, out and total strength greater than 10,000; and, finally, the right-hand portion of all the distributions shows that only less than 1% of all sectors have an in, out and total strength greater than 100,000. Therefore, on the contrary to the degree distributions observed, the in, out and total strength distributions for sectors in the IPN are all positively skewed.

The findings in Acemoglu et al. (2012) and Carvalho (2014) suggest that in production networks like the IPN, where sectors are both highly connected and asymmetrically connected, a local shock is able to propagate through the whole economy and generate a sizeable global disturbance. However, the size and the spread of the shock depend also on the weight and the position within the network of the affected sectors: the more a locked sector is big and central within the network, the higher the aggregate loss are. Moreover, as lockdowns are selective (governments mandate that certain industries deemed ‘essential’ should remain open whereas others, especially the riskiest from an epidemiological perspective, are locked), understanding the relevance of sectors within the production network is of foremost importance to design predictive tools and better inform regulators on how to dampen aggregate variability and reduce the likelihood of systemic risk due to lockdowns.

In this vein, Tables 1 and 2 show the key sectors in the IPN in terms of nodes strength and nodes centrality. The computation is made according to two methods, the PageRank algorithm (Brin and Page 1998) and the hypothetical extraction (HE) technique (Miller and Lahr 2001).^3^ The unequal strength distributions depicted in Fig. 1 suggests the presence of hub-like sectors. In fact, as shown in Table 1, the IPN is dominated in terms of strength (defined as the euro goods that flow through a sector) by a few industries that could act as global propagators in the network. According to the strength rankings, the core sectors are construction (F), which is the largest sector in terms of in and total strength, wholesale trade (46) (the largest in terms of out-strength), food products (10_12), financial services (64), machinery and equipment (28), legal and accounting (69_70), and electricity and gas (D).

**Table 1:** Sectors strength (millions of euro) and centrality

IN-strength		OUT-strength		TOT-strength		PageRank
F	117,402.5	46	83,997.8	F	186,886.8	F
10_12	107,073.4	64	73,418.9	10_12	173,778.4	46
46	84,019.2	F	69,484.3	46	168,017	28
28	79,303.4	69_70	68,758.8	28	125,117.8	10_12
D	59,151.4	10_12	66,704.9	D	123,628.5	I
13_15	56,370.7	D	64,477.1	25	111,134.9	86
29	50,223.2	49	63,296.2	49	108,160.1	29
I	50,191.6	25	61,146.5	64	101,401	80_82
25	49,988.3	80_82	56,280.6	69_70	96,345.4	O
86	49,345.6	L	55,636.9	24	94,084.9	47
47	48,284.6	24	51,863.2	13_15	93,718.6	25
49	44,863.9	52	50,822.0	80_82	90,451.8	49
24	42,221.7	20	49,983.5	20	87,902.0	13_15
O	39,116.5	28	45,814.4	52	87,535.9	D
20	37,918.5	1	43,753	L	83,290.4	24
52	36,713.9	13_15	37,347.9	29	78,780.6	20
80_82	34,171.2	62_63	36,379.2	I	74,050.6	31_32
19	32,828.5	22	32,153.5	47	70,859.3	45
22	29,121.2	B	30,614.6	1	66,607.6	L
31_32	28,305.9	29	28,557.4	62_63	63,397.9	22

Full list and sector codes definitions are shown in Table A.1 in Supplementary appendix

**Table 2:** Key sectors according to hypothetical extraction

Sector codes	Absolute LVA	Relative LVA	Direct LVA	Indirect LVA	Direct share	Indirect share
L	− 227,218	− 14.92	− 207,395	− 19,823.2	91.28	8.72
46	− 139,142	− 9.14	− 82,416.2	− 56,726.2	59.23	40.77
O	− 129,661	− 8.51	− 100,661	− 28,999.5	77.63	22.37
F	− 120,742	− 7.93	− 65,598.6	− 55,143.4	54.33	45.67
47	− 118,324	− 7.77	− 79,303.2	− 39,021.2	67.02	32.98
86	− 105,986	− 6.96	− 77,702.4	− 28,283.9	73.31	26.69
I	− 93,851.3	− 6.16	− 57,278.1	− 36,573.2	61.03	38.97
10_12	− 80,056.8	− 5.26	− 27,913.3	− 52,143.4	34.87	65.13
28	− 79,191.1	− 5.2	− 36,207.8	− 42,983.3	45.72	54.28
P	− 71,841.8	− 4.72	− 63,365.9	− 8475.93	88.20	11.80
64	− 71,096.7	− 4.67	− 56,140.7	− 14,955.9	78.96	21.04
49	− 69,468.5	− 4.56	− 45,486.2	− 23,982.3	65.48	34.52
69_70	− 64,426.2	− 4.23	− 47,911.3	− 16,514.9	74.37	25.63
80_82	− 53,462.9	− 3.51	− 29,890	− 23,572.9	55.91	44.09
25	− 52,537.9	− 3.45	− 29,982.7	− 22,555.3	57.07	42.93
52	− 48,617.6	− 3.19	− 30,549.1	− 18,068.4	62.84	37.16
13_15	− 45,572.5	− 2.99	− 24,437.4	− 21,135.1	53.62	46.38
62_63	− 44,714.9	− 2.94	− 30,101.5	− 14,613.4	67.32	32.68
D	− 44,678.7	− 2.93	− 23,966.4	− 20,712.3	53.64	46.36
29	− 41,876.6	− 2.75	− 13,791	− 28,085.6	32.93	67.07

Full list and sector codes definitions are shown in Table A.1 in Supplementary appendix. Absolute LVA is in millions of euro

Regarding the centrality within the network, the PageRank algorithm revels that the most influential sectors, namely the nodes that are central in the network and linked with other central nodes, are construction (F), wholesale trade (46), machinery and equipment (28), food products (10_12), and accommodation and food services (I).

The strength and PageRank centrality measures are computed using the inter-industry transactions matrix. Therefore, these measures are based on the supply intermediate chains and do not account for final demand. Yet, the lockdowns involve both supply and demand. Hence, in order to unveil the potential impact of total sectoral lockdowns we employ the HE technique as this is widely used in the input–output literature to identify key sectors (for a complete review and insights see Dietzenbacher and Lahr 2013 and Miller and Blair 2009). Specifically, the HE quantifies how much the output of an *n*-sectors economy would decrease if a particular industry were not present or, in our case, locked.

We next briefly explain how the HE technique is implemented in an input–output scheme. Consider an economy with $n$ industries and denote the interindustry flows by the $n\times n$ transaction matrix $\mathbf{Z}$.^4^ Let $\mathbf{f}$ be the vector of industry final demands and $\mathbf{x}$ the vector of industry gross output. The accounting equations are given as $\mathbf{x}=\mathbf{Z}\mathbf{i}+\mathbf{f}$, where $\mathbf{i}$ is the summation vector, i.e. a vector of all ones. Define the direct input coefficients as the ratio of input supplied by $i$ and bought by $j$ over the gross output of sector $j$ as ${a}_{ij}={z}_{ij}/{x}_{j}$, which is the typical element of the economy’s direct requirements matrix $\mathbf{A}$, also known as the technical coefficients matrix. Considering that, $\mathbf{A}=\mathbf{Z}{\widehat{\mathbf{x}}}^{-1}$ we can substitute $\mathbf{A}\mathbf{x}=\mathbf{Z}\mathbf{i}$ in the accounting equations to get $\mathbf{x}=\mathbf{A}\mathbf{x}+\mathbf{f}$. Solving for $\mathbf{x}$ yields:

1 $$\mathbf{x}={(\mathbf{I}-\mathbf{A})}^{-1}\mathbf{f}=\mathbf{L}\mathbf{f}$$

where $\mathbf{I}$ is the identity matrix and ${\mathbf{L}\equiv (\mathbf{I}-\mathbf{A})}^{-1}$ is the Leontief inverse or multiplier matrix, which makes clear the direct and indirect dependence of each of gross outputs on the values of each of the final demand. Extracting industry $k$ requires that the *k*th row and column of the $\mathbf{A}$ matrix are set equal to zero. We define this matrix by ${\mathbf{A}}^{*}$. Equally, the final demand for goods and services provided by industry $k$ is set to zero, i.e.${f}_{k}=0$, which gives the new final demand vector ${\mathbf{f}}^{\boldsymbol{*}}$. Thus, the estimated new vector of sector gross outputs will be:

2 $${\mathbf{x}}^{*}={(\mathbf{I}-{\mathbf{A}}^{\mathbf{*}})}^{-1}{\mathbf{f}}^{\boldsymbol{*}}$$

The change before and after extraction is equal to the difference ${\mathbf{s}}^{^{\prime}}=(\mathbf{x}-{\mathbf{x}}^{\mathbf{*}})$. To express this change in GDP terms we simply pre-multiply Eq. (2) by the value added coefficients matrix $\widehat{\mathbf{V}}$, i.e. a diagonal matrix, of which the typical element on the main diagonal, ${v}_{j}^{s}/{x}_{j}^{s}$, is the value added coefficient of industry *j* in country *s*. This leads to:

3 $${\mathbf{v}}^{*}={\widehat{\mathbf{V}}(\mathbf{I}-{\mathbf{A}}^{\mathbf{*}})}^{-1}{\mathbf{f}}^{\boldsymbol{*}}$$

Finally, the absolute change in value added is derived by the difference ${\mathbf{s}}^{^{\prime}}=(\mathbf{v}-{\mathbf{v}}^{\mathbf{*}})$, or in relative terms by the ratio $(\mathbf{v}-{\mathbf{v}}^{\mathbf{*}})/\mathbf{v}$.

We studied the impact on value added of single sector extractions or lockdowns, i.e. we extracted—or locked—one at a time all the 63 sectors and computed the new value added according to Eq. (3). Table 2 reports the top 20 sectors ranked by the total value added locked. The results suggest that the greater lock in value added (LVA) would be triggered by the lockdown of real estate activities (L), wholesale trade (46), public administration (O), construction (F), and retail trade (47). As explained in the next section, the IPMD locked almost all the above-mentioned industries with the only exception of public administration (O). In general, the IPMD locked about 40% of the 20 key sectors reported in Table 2. Columns 4 and 5 of Table 2 split out the total absolute LVA into direct—i.e. aggregate value added minus sector’s value added—and indirect—i.e. aggregate locked value added minus sector’s value added—locked value added. The LVA due to the real estate (L) lockdown is almost entirely imputable to a direct impact. Whereas other industries like wholesale trade (46), construction (F), food products (10_12), machinery and equipment (28) produce a LVA propagating mainly through indirect value chains. This result is not surprising since such sectors are the main sellers and purchasers of intermediates in the IPN, as shown by the in and out-strengths ranking, and are indirectly well connected with other core sectors, as captured by the PageRank centrality measure.

## A measure of locked GDP

In this section we attempt to quantify the amount of Italian GDP locked by the IPMD and explore some policy alternatives under different scenarios of epidemiological risk. It should be stressed that we here refer to the value added locked, and not to the real economic impact of Covid-19 lockdown. Actual losses will depend on many non-forecastable factors such as second round impacts, postponed consumption and investment, and so on, elements that are not contemplated in this study.

### Methodology and data description

The IPMD defines and lists the industries deemed ‘essential’ that are not subject to lockdown. The list of ‘essential’ industries includes NACE industrial classification codes at 2-digit, equally to the industrial classification provided by the ISTAT’s SIOT. As discussed in the previous section, the HE method would be well suited theoretically and practically to simulate sectorial lockdowns of non-essential industries. However, the IPMD’s list does not always involve entire 2-digit sectors. Rather, sectors are partially affected, i.e. just some establishments within the sectors are subject to lockdown. Hence, the full HE technique is not appropriate to quantify the amount of locked GDP. Nevertheless, according to Dietzenbacher and Lahr (2013), the HE method can be expanded and generalized to a large variety of applications, among which the case of partial capacity constraints. Notably, Dietzenbacher and Lahr (2013) assume that an industry (say $k$) consists of identical establishments, one of which ceases to exist so that the industry’s capacity reduces. In this case, a total extraction (nullification) will not occur, simply the intermediate and final deliveries sold by this industry decrease by a percentage $\alpha \cdot 100\%$. Hence, the new technical coefficient will be equal to ${a}_{kj}^{*}={z}_{kj}^{*}/{x}_{j}={\left(1-\alpha \right)z}_{kj}^{*}/{x}_{j}=(1-\alpha ){a}_{kj}$ and the new final demand will be equal to ${f}_{k}^{*}=(1-\alpha ){f}_{k}$. Giammetti (2020) recently used this method known as partial hypothetical extraction (PE) in a Brexit study to partially lock trade between the UK and the EU. Similarly, here we employ the PE technique to partially lock the production processes of some industries.

In order to compute the share of sector $i$ value added locked by the IPMD we divided the value added of the establishments subjected to lockdown by the sum of all the establishments included in sector $i$, i.e. sector $i$’s value added.^5^ This share corresponds to ${\alpha }_{i}$. Then, we multiply $(1-{\alpha }_{i}$) times all the elements of the *i*th row and column of $\mathbf{A}$, and times the *i*th element of vector $\mathbf{f}.$

To better understand this, consider a three sectors IO model, the three sectors being open (o), totally locked (t) (i.e., ${\alpha }_{t}=1$) and partially locked (p). The $\mathbf{A}$ matrix and ${\varvec{f}}$ vector for this model will be given as:

4 $$\mathbf{A}=\left[\begin{array}{ccc}{a}_{oo}& {a}_{ot}& {a}_{op}\\ {a}_{to}& {a}_{tt}& {a}_{tp}\\ {a}_{po}& {a}_{pt}& {a}_{pp}\end{array}\right] \quad {\varvec{f}}=\left[\begin{array}{c}{f}_{o}\\ {f}_{t}\\ {f}_{p}\end{array}\right]$$

where ${a}_{ij}$ gives the units of intermediate goods produced in sector $i$ needed to produce one unit of the good in sector $j$, or alternatively, the input demand in sector $j$ for intermediate goods produced in sector $i$. Similarly, ${f}_{i}$ is the quantity of final product produced in sector $i$ demanded domestically. So, in this three-sector example, the elements $\mathbf{A}$ and ${\varvec{f}}$ involved in a lockdown scenario are all but the diagonal element ${a}_{oo}$ and the final demand ${f}_{o}$. The modified $\mathbf{A}$ and ${\varvec{f}}$ reported in the following Eq. (5)

5 $$\mathbf{A}=\left[\begin{array}{ccc}{a}_{oo}& 0& {{{\varvec{a}}}_{{\varvec{o}}{\varvec{p}}}}^{\boldsymbol{*}}\\ 0& 0& 0\\ {{{\varvec{a}}}_{{\varvec{p}}{\varvec{o}}}}^{\boldsymbol{*}}& 0& {{{\varvec{a}}}_{{\varvec{p}}{\varvec{p}}}}^{\boldsymbol{*}}\end{array}\right] \quad {\varvec{f}}=\left[\begin{array}{c}{f}_{o}\\ 0\\ {{{\varvec{f}}}_{{\varvec{p}}}}^{\boldsymbol{*}}\end{array}\right]$$

are then plugged into Eq. (3) to compute the locked GDP for each sector.

So far, we have not considered the epidemiological risk linked to sectoral production activities. In what follows, we use an index of proximity for workers operating in different sectors of the Italian economy to estimate the LVA under different lockdown policies. This sectoral physical proximity index (PPI), taken from Table 1 of Barbieri et al. (2020), is used to divide the sectors into four classes of risk.^6^

**Fig. 2:**
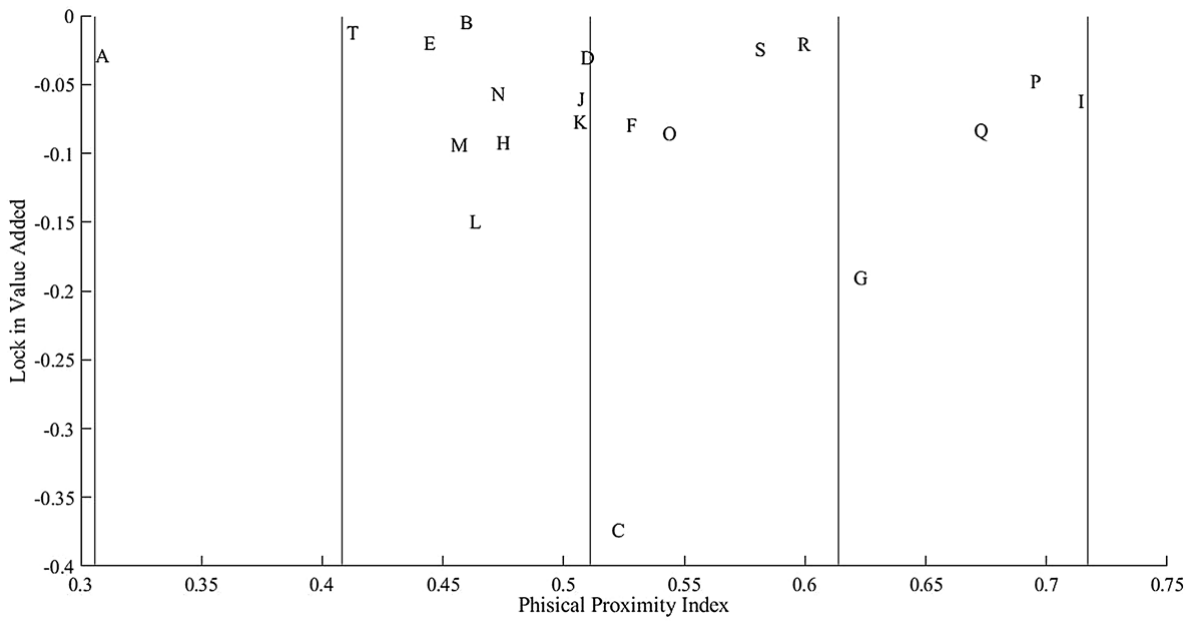
Epidemiological and economic sectoral risk

Figure 2 shows the sectors divided by their PPI on the *x*-axis and the LVA generated by their lockdown on the *y*-axis.^7^ Hence, the left (right)-hand portion of the figure shows the sectors that are less (more) risky from an epidemiological perspective. Moving from top to bottom, LVA is higher. The sector agriculture (A) has the lowest PPI and its lockdown would lock about a 3% of aggregate GDP. The second class includes sectors of medium-low epidemiological risk. Among these, real estate (L), professional activities (M), transportation (H), and finance (K) are economically relevant. The third and fourth classes include sectors of medium-high to high epidemiological risk. Apparently, there might be a trade-off between economic and epidemiological risk for sectors like manufacturing (C), construction (F), public administration (O), wholesale and retail trade (G), and accommodation and food services (I).^8^ On the other hand, there might be no trade-off for sectors like sports and recreational (R) and other services (S), that have a high PPI but would have a limited economic impact in case of a lockdown.

Table 3 summarizes the set of different lockdown policies we tested ranked by epidemiological risk. We assume different locking thresholds for each sectors' class. For example, in the low-risk scenario, low-risk sectors are open whereas medium-low, medium-high and high-risk sectors are respectively locked at 50, 70, and 100%. It should be noted that while the IPMD distinguishes between ‘essential’ sectors that can remain open and sectors partially or fully subjected to lockdown, our scenarios provide that all sectors included in an epidemiological risk class are equally subject to the same percentage of lockdown. As shown in Table A.2 in the Supplementary appendix, on average the IPMD policy is comparable with our Medium-risk scenario, as it orders the opening of low-risk sectors and a lockdown for medium-low, medium-high, and high-risk sectors of about 0.35, 0.5, and 0.5 respectively.

**Table 3:** Counterfactual scenarios

	Low-risk sectors lockdown	Medium-low risk sectors lockdown	Medium-high risk sectors lockdown	High-risk sectors lockdown
Low-risk scenario	0	0.5	0.7	1^a^
Medium-risk scenario	0	0	0.5	1^b^
High-risk scenario	0	0	0	0.5

^a^The high-risk sectors class includes health (Q), wholesale and retail trade (G), and education (P). Since health cannot be locked down during a pandemic, and education can be provided remotely, we assume that these two sectors are open in all the three scenarios. Further, we assume that as people need food and beverage, the sector wholesale and retail trade (G) remains half open, i.e. subjected to a 0.5 lockdown in all the three scenarios. Hence, the percentage closure of high-risk sectors refers only to the accommodation and food services (I) sector.

^b^Note that Table 1 in Barbieri et al. (2020) provides information for 21 aggregate sectors. Therefore, in Fig. 2 we grouped our 63 2-digit sectors in 20 aggregate sectors. Note that sector number 21 representing international organizations (U), reported in Barbieri et al. (2020), is not present in the ISTAT SIOT’s tables.

## Results

The output of our simulations is presented in Table 4. According to our findings, the lockdown imposed by the IPMD would have locked about the 52% of total GDP. The magnitude of this result is consistent with the estimates of the Bank of Italy (2020); ours are slightly higher as we account for indirect impacts. Indeed, as shown in Table A.2, the amount of value added directly subject to lockdown according to the IPMD list is about 37% of total GDP, i.e. the 70% of total locked value added (LVA). The remaining 30% is locked within indirect value chains.

**Table 4:** Scenario analysis results in absolute (millions of euro) and relative locked-value-added (LVA)

	Absolute LVA	Relative LVA	Direct LVA	Indirect LVA
IPMD scenario	− 799,704.15	− 52.51	70.63	29.37
Low-risk scenario	− 970,947.89	− 63.76	75.81	24.19
Medium-risk scenario	− 636,754.01	− 41.81	54.16	45.84
High-risk scenario	− 222,970.34	− 14.64	52.91	47.09

**Fig. 3:**
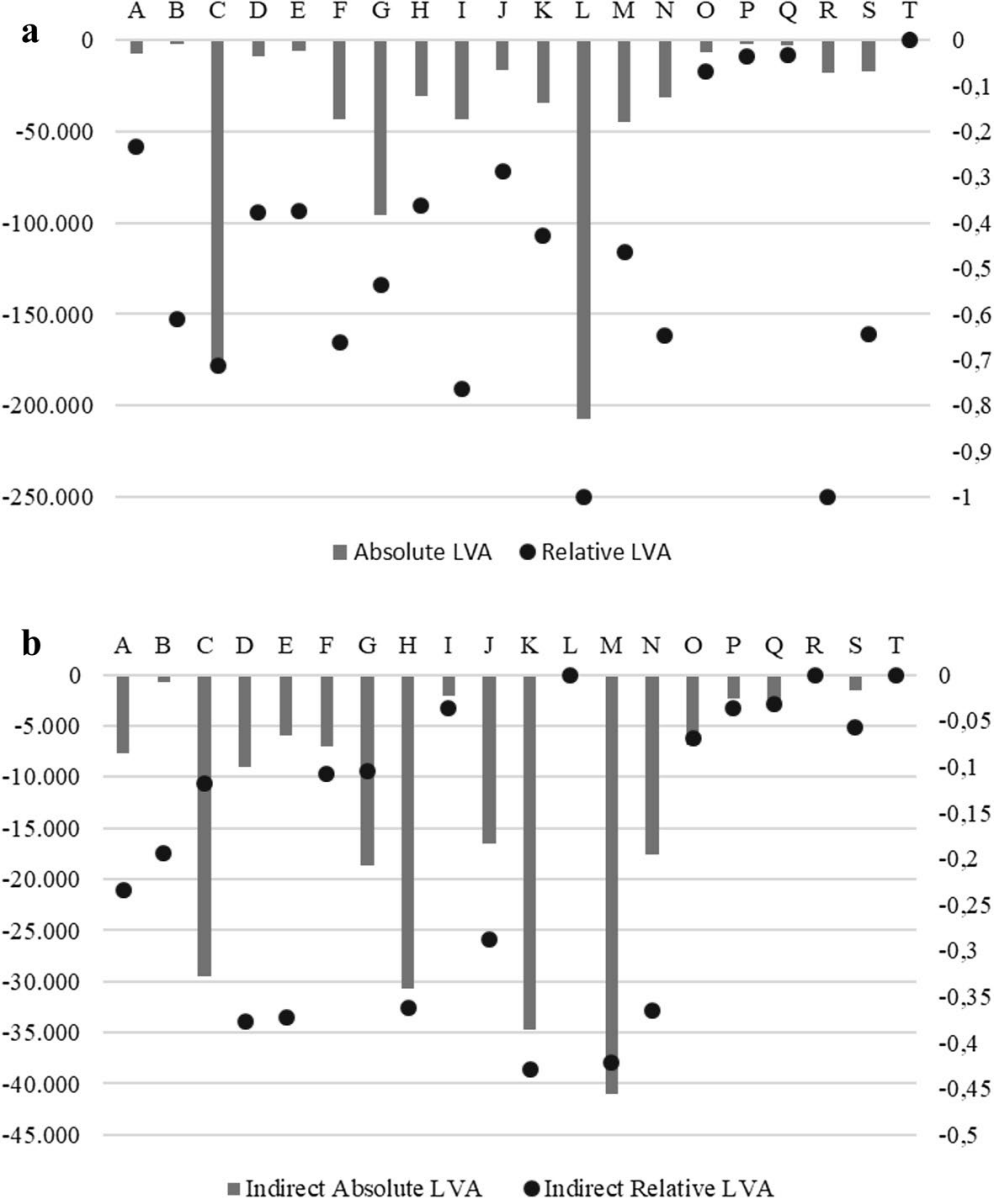
**a** Total absolute and relative LVA at sectoral level. **b** Indirect absolute (millions of euro) and relative LVA at sectoral level

Figure 3a, b show the absolute and relative LVA at sectoral level (2-digit sectors results are provided in Table A.3, in the appendix). The sectors most affected by the IPMD are manufacturing (C), construction (F), accommodation and food services (I), sports and recreational (R). However, if we leave out the LVA determined directly by the IPMD and look at the value added locked by the input–output relationships (Fig. 3b), we find that even sectors that are not subjected to lockdown are significatively affected by the IPMD. Among these, for example financial (K) and professional (M) services, transportation (H), information (J), energy and gas (D), water and waste (E), and agriculture (A).

As mentioned earlier, the IPMD list distinguishes between ‘essential’ and not ‘essential’ industries without taking into account the input–output structure of the economy. However, as shown by Fig. 3a, b, stopping the production activities of a sector leads to a drop in input and output delivered and purchased by the locked sector. Therefore, as an alternative to IPMD, sectors could be distinguished in terms of epidemiological risk and partial locked based on the risk class to which they belong. This is what we propose in the three scenarios reported in Table 3. Clearly, moving from the safer scenario to the riskier one, LVA decreases. Notably, as shown in Table 4, in a low risk scenario the total LVA would be higher than the IPMD scenario. Whereas, the medium risk scenario, which is comparable in terms of epidemiological risk with the IPMD scenario, would lead to a lower LVA. Finally, the high-risk scenario gives us an idea of the economic relevance of the wholesale and retail trade (G) sector, and accommodation and food services (I) industry. Closing 50% of these two sectors, leaving the rest of the economy open, would still result in a significant LVA.

## Conclusion

The recent Covid-19 pandemic has forced governments of all countries to adopt social distancing restrictions and to lock part of production activities. The extent of these measures has varied substantially across countries depending on the intensity of the pandemic as well as on governments’ preferences about the trade-off between public health and output losses. While some countries are still facing serious difficulties in keeping the spread of the virus in check, the emergency is over in many others. However, also the latter should use this period of calm to prepare themselves for potential other waves of resurgence of infections that could materialize in the forthcoming months. This means making contingent plans that, while being effective in containing the spread of the virus, do not generate excessive output losses. Indeed, the Covid-19 crisis has hit economies that had not yet fully absorbed the effects of the 2008 great recession, and therefore it might have dramatic implications on the rise of unemployment, the exacerbation of inequalities, the explosion of public and private debt. Therefore, it is of major importance that future governments’ choices facing Covid-19 pandemic will also be guided by the minimization of the value added losses. This in turn requires taking into consideration that production structures are characterized by complex value chains and that the interconnections between sectors have important implications for the propagation of the shocks across the economy (e.g., Acemoglu et al. 2012).

As emphasized in the Introduction, even though the last few months have seen the emergence of a fast-growing literature studying the effects of Covid-19 related governments’ restrictions on the economy, the complementarities and more generally the links between production sectors are rarely included in analysis (Caracciolo et al. 2020). In this paper we tried to move in this direction by performing a quantitative assessment of the role of the domestic value chain in transmitting the economic impact of Covid-19 in Italy. We first analyzed the Italian production network and found that sectors are both highly connected and asymmetrically connected. Hence, a local shock due to lockdown policy propagates through the whole economy and generates a sizeable global disturbance. This finding is amplified by the fact that the Italian domestic chain is dominated by a few hub sectors that act as general suppliers delivering inputs to many or all other sectors.

We found that the stop of the production processes of many of these key sectors following the IPMD rules has led to a drop in input and output that, in turn, have generated a significant lock of circulating value added. Notably, our results suggest that the lockdown measures taken by Italian government would have locked about the 52% of total GDP, 30% of which has been locked within indirect value chains.

Our results confirm the importance of value chain analysis in investigating how the economy adjusts to dislocation and destruction of parts of its productive capacity. The scenario analysis presented confirms that taking into account the input–output structure of the economy when defining lockdown and reopening plans is key. Indeed, we tested a set of different policies in order to provide results that might help policy makers in defining the criteria for lockdown and reopening plans. As we have stressed above, for each level of contagion for workers and communities, policies should aim at minimizing the economic losses. Regarding the latter, we showed that some sectors have more weight in the production process: they are central within the production network. At the same time, sectors also differ in terms of contagion risk in at least two dimensions reflecting the specificities of the tasks performed by their employees: the physical proximity that the tasks require, and the teleworking. In this work, we have employed a proxy for the physical proximity from colleagues, whereas we did not account for the physical distance from customers and the ability to perform work from home. While the IPMD has distinguished the industries ‘essential’ and ‘not essential’, the idea at the base of this work is that lockdown and reopening plans should prioritize sectors with higher economic benefits and less risk for workers.

Another aspect that should not be overlooked is that the IPMD has hit people with different income brackets asymmetrically. The rules contained in such government decree imply that the probability of being subject to lockdown and not being able to work is higher for workers with lower wages than that one of those with wages in the highest quartile of the distribution (Palomino et al. 2020). Hence, as low-wage workers are also more numerous in sectors with low value added (Fana et al. 2020), a reopening strategy giving priority to the sectoral value added would lead to greater benefits to high-wage workers. This suggests that measures capable of containing the value added losses should be accompanied by redistributive fiscal policies sustaining the wages and employment of the economically weaker parts of the population.

Finally, one should not forget that governments and their policies are not immune to political economy issues also during a pandemic. While we have not discussed the role of special interest politics in shaping the public health related policy restrictions, we think this is an interesting avenue for future research.

## Electronic supplementary material

Below is the link to the electronic supplementary material.

Supplementary file1 (DOCX 34 kb)

## Footnotes

1 It should be stressed that we here refer to the value added locked, and not to the real economic impact of Covid-19 lockdown. Actual losses will depend on many non-forecastable factors such as second round impacts, postponed consumption and investment, and so on, elements that are not contemplated in this study. For more details see Sect. 3.

2 To save space, in what follows we just report some basic network statistics usually employed to study production networks, such as the degree and strength distribution. For details, definitions, and discussion on the measures employed see Giammetti et al. (2020).

3 It should be noted that a relevant strand of the literature, rather than emphasize the role played by a single central sector, performs a key cluster analysis to identify industry blocks and visualize the resulting fundamental inter-industry structure (see Garbellini and Wirkierman 2014).

4 Matrices are indicated by bold capitals, vectors by bold lower cases and scalars by italic lower cases. Diagonal matrices are indicated by a hat over the vector containing the elements on the main diagonal. Primes indicate transposition.

5 The IPMD refers to 2, 4, and 6-digit industries whereas the ISTAT’s SIOT tables are in 2-digit. Therefore, to compute the value added share of a 2-digit sector locked by the IPMD we employ 6-digit data from the Enterprises economic indicators database provided by ISTAT.

6 The four intervals are computed with the standard class interval formula: $\frac{highest \, value-lowest \, value}{number \, of \, classes}$.

7 Note that Table 1 in Barbieri et al. (2020) provides information for 21 aggregate sectors. Therefore, in Fig. 2 we grouped our 63 2-digit sectors in 20 aggregate sectors. Note that sector number 21 representing international organizations (U), reported in Barbieri et al. (2020), is not present in the ISTAT SIOT’s tables.

8 We do not comment on the health sector (Q) as, although it is epidemiologically risky, it must remain open during a pandemic.
