# Supplementary material for: The Italian value chain in the pandemic: the input–output impact of Covid-19 lockdown
Source: J. Ind. Bus. Econ. 2020 Jul 6;47(3):483–97. doi: 10.1007/s40812-020-00164-9 (PMC7335925; doi:10.1007/s40812-020-00164-9)
Supplement: Supplementary file 1 — Supplementary file1 (DOCX 34 kb) [file 40812_2020_164_MOESM1_ESM.docx]

**Appendix**

| Codes | Sectors Description | In-Strength | Out-Strength | Tot-Strength | Pageranks | HE |
| --- | --- | --- | --- | --- | --- | --- |
| 01 | Crop and animal production | 22854,57 | 43753 | 66607,57 | 0,015398 | -40286,5 |
| 02 | Forestry | 380,18 | 1831,34 | 2211,52 | 0,00253 | -2063,67 |
| 03 | Fishing | 690,53 | 898,85 | 1589,38 | 0,002975 | -1438,51 |
| B | Mining and quarrying | 3875,52 | 30614,63 | 34490,15 | 0,004861 | -6368,34 |
| 10_12 | Food Product | 107073,4 | 66704,97 | 173778,4 | 0,042393 | -80056,8 |
| 13_15 | Textiles | 56370,74 | 37347,9 | 93718,64 | 0,020399 | -45572,5 |
| 16 | Wood and Cork | 9401,69 | 12986,14 | 22387,83 | 0,007325 | -9503,37 |
| 17 | Paper Products | 16936,26 | 19122,08 | 36058,34 | 0,009897 | -13710,1 |
| 18 | Printing and Media | 6802,22 | 10828,01 | 17630,23 | 0,007372 | -9107,86 |
| 19 | Petroleum Products | 32828,49 | 21354,43 | 54182,92 | 0,013071 | -13871,3 |
| 20 | Chemicals | 37918,53 | 49983,51 | 87902,04 | 0,019299 | -28450,7 |
| 21 | Pharmaceutical | 15638,93 | 16320,67 | 31959,6 | 0,011577 | -17501,2 |
| 22 | Rubber and Plastic | 29121,17 | 32153,48 | 61274,65 | 0,017938 | -28022,1 |
| 23 | Other Non-metallic mineral | 19318,15 | 23376,57 | 42694,72 | 0,011364 | -19847,5 |
| 24 | Basic Metals | 42221,73 | 51863,16 | 94084,89 | 0,019883 | -27174,2 |
| 25 | Metal products | 49988,31 | 61146,54 | 111134,9 | 0,026129 | -52537,9 |
| 26 | Electronics and Computers | 13429,13 | 14097,6 | 27526,73 | 0,010379 | -15886,1 |
| 27 | Electrical Equipment | 28082,56 | 24467,04 | 52549,6 | 0,016602 | -25288,4 |
| 28 | Machinery & Equipment | 79303,39 | 45814,43 | 125117,8 | 0,043636 | -79191,1 |
| 29 | Motor vehicles | 50223,19 | 28557,36 | 78780,55 | 0,033432 | -41876,6 |
| 30 | Transport equipment | 17176,52 | 9626,33 | 26802,85 | 0,013592 | -16855,8 |
| 31_32 | Furniture & other manufac. | 28305,88 | 16848,23 | 45154,11 | 0,019034 | -29358,3 |
| 33 | Installation of machinery | 10364,92 | 9730,95 | 20095,87 | 0,011239 | -15529,7 |
| D | Electricity & Gas | 59151,36 | 64477,11 | 123628,5 | 0,020253 | -44678,7 |
| 36 | Water Collection Activities | 5760,51 | 5461,76 | 11222,27 | 0,006177 | -9793,21 |
| 37_39 | Waste Collection Activities | 16608,92 | 23109,43 | 39718,35 | 0,013414 | -20401,2 |
| F | Construction | 117402,5 | 69484,33 | 186886,8 | 0,051027 | -120742 |
| 45 | Wholesale and retail trade | 21556,24 | 14484,18 | 36040,42 | 0,018988 | -31759,4 |
| 46 | Wholesale trade | 84019,21 | 83997,82 | 168017 | 0,046702 | -139142 |
| 47 | Retail trade | 48284,55 | 22574,72 | 70859,27 | 0,026752 | -118324 |
| 49 | Land & Pipeline transport | 44863,92 | 63296,21 | 108160,1 | 0,025354 | -69468,5 |
| 50 | Water transport | 7634,02 | 3388,2 | 11022,22 | 0,009505 | -9110,93 |
| 51 | Air transport | 8498,76 | 7673,18 | 16171,94 | 0,010952 | -7540,98 |
| 52 | Warehousing | 36713,87 | 50822,04 | 87535,91 | 0,01745 | -48617,6 |
| 53 | Postal | 3071,01 | 6078,81 | 9149,82 | 0,004313 | -5829,72 |
| I | Accommodation & Food serv. | 50191,58 | 23859,06 | 74050,64 | 0,041508 | -93851,3 |
| 58 | Publishing Act. | 5768,6 | 3640,29 | 9408,89 | 0,006184 | -7354,18 |
| 59_60 | Media Production | 10037,76 | 9289,58 | 19327,34 | 0,008087 | -11969,2 |
| 61 | Telecom | 19974,43 | 22586,98 | 42561,41 | 0,010836 | -28409,8 |
| 62_63 | Computer Programming | 27018,63 | 36379,23 | 63397,86 | 0,016194 | -44714,9 |
| 64 | Financial Services | 27982,15 | 73418,88 | 101401 | 0,011898 | -71096,7 |
| 65 | Insurance | 16906,8 | 9752,07 | 26658,87 | 0,006454 | -22792 |
| 66 | Auxiliary Financial Serv. | 11626,16 | 25762,52 | 37388,68 | 0,007784 | -23300,3 |
| L | Real Estate | 27653,47 | 55636,9 | 83290,37 | 0,018121 | -227218 |
| 69_70 | Legal and Accounting | 27586,62 | 68758,81 | 96345,43 | 0,017402 | -64426,2 |
| 71 | Architectural and engineering act. | 14047,19 | 25426,76 | 39473,95 | 0,01085 | -26937,8 |
| 72 | Scientific Research | 3831,23 | 2959,71 | 6790,94 | 0,005124 | -18420,6 |
| 73 | Advertising and market research | 12436,43 | 16576,53 | 29012,96 | 0,016486 | -13993,7 |
| 74_75 | Other professional activities | 8884,6 | 19078,45 | 27963,05 | 0,008593 | -19199,2 |
| 77 | Rental and leasing activities | 7334,64 | 14060,17 | 21394,81 | 0,007891 | -12893,8 |
| 78 | Employment activities | 1163,67 | 9463,94 | 10627,61 | 0,003196 | -9838,93 |
| 79 | Travel agency & tour operator | 10058,99 | 5506,57 | 15565,56 | 0,012858 | -9665,17 |
| 80_82 | Security, landscape, administrative | 34171,18 | 56280,57 | 90451,75 | 0,027828 | -53462,9 |
| O | Public administration | 39116,48 | 18293,43 | 57409,91 | 0,027347 | -129661 |
| P | Education | 11375,21 | 6189,71 | 17564,92 | 0,011869 | -71841,8 |
| 86 | Human health activities | 49345,6 | 13407,68 | 62753,28 | 0,038517 | -105986 |
| 87_88 | Residential care & social work | 10396,46 | 7680,83 | 18077,29 | 0,016028 | -20807,4 |
| 90_92 | Entertainment, culture, betting | 14414,56 | 10987,18 | 25401,74 | 0,00984 | -17817,2 |
| 93 | Sports activities | 9889,66 | 9528,16 | 19417,82 | 0,008091 | -13541 |
| 94 | Membership organisations | 5790,24 | 4021 | 9811,24 | 0,006214 | -8912,45 |
| 95 | Repair of computers | 1383,63 | 1682,17 | 3065,8 | 0,003381 | -2849,55 |
| 96 | Other personal services | 8367,91 | 4122,59 | 12490,5 | 0,007829 | -26717,7 |
| T | Activities of households as employers | 0 | 0,02 | 0,02 | 0,002381 | -18440,2 |

**Table A.1:** Key sectors according to strength PageRank centrality and Hypothetical Extraction.

| Codes | Sectors Description | Firms locked | Workers locked | Value added locked | Absolute value added locked |
| --- | --- | --- | --- | --- | --- |
| 1 | Crop and animal production | 0,00 | 0,00 | 0,00 | 0,00 |
| 2 | Forestry | 0,00 | 0,00 | 0,00 | 0,00 |
| 3 | Fishing | 0,00 | 0,00 | 0,00 | 0,00 |
| B | Mining and quarrying | 0,98 | 0,80 | 0,42 | 1522,24 |
| 10_12 | Food Product | 0,00 | 0,00 | 0,01 | 288,56 |
| 13_15 | Textiles | 0,96 | 0,95 | 0,93 | 22833,41 |
| 16 | Wood and Cork | 0,00 | 0,00 | 0,00 | 0,00 |
| 17 | Paper Products | 0,42 | 0,26 | 0,19 | 1065,96 |
| 18 | Printing and Media | 0,00 | 0,00 | 0,00 | 0,00 |
| 19 | Petroleum Products | 0,00 | 0,00 | 0,00 | 0,00 |
| 20 | Chemicals | 0,21 | 0,18 | 0,20 | 2349,44 |
| 21 | Pharmaceutical | 0,00 | 0,00 | 0,00 | 0,00 |
| 22 | Rubber and Plastic | 0,66 | 0,64 | 0,64 | 8075,36 |
| 23 | Other Non-metallic mineral | 0,95 | 0,89 | 0,85 | 8387,59 |
| 24 | Basic Metals | 1,00 | 1,00 | 1,00 | 8429,48 |
| 25 | Metal products | 0,93 | 0,89 | 0,87 | 26088,36 |
| 26 | Electronics and Computers | 0,39 | 0,42 | 0,45 | 3670,19 |
| 27 | Electrical Equipment | 0,69 | 0,63 | 0,62 | 6853,61 |
| 28 | Machinery & Equipment | 0,76 | 0,78 | 0,77 | 27860,11 |
| 29 | Motor vehicles | 1,00 | 1,00 | 1,00 | 13790,98 |
| 30 | Transport equipment | 1,00 | 1,00 | 1,00 | 7400,38 |
| 31_32 | Furniture & other manufac. | 0,59 | 0,69 | 0,64 | 8779,43 |
| 33 | Installation of machinery | 0,57 | 0,49 | 0,45 | 3886,65 |
| D | Electricity & Gas | 0,00 | 0,00 | 0,00 | 0,00 |
| 36 | Water Collection Activities | 0,00 | 0,00 | 0,00 | 0,00 |
| 37_39 | Waste Collection Activities | 0,00 | 0,00 | 0,00 | 0,00 |
| F | Construction | 0,71 | 0,60 | 0,55 | 36336,71 |
| 45 | Wholesale and retail trade | 0,19 | 0,27 | 0,44 | 7493,37 |
| 46 | Wholesale trade | 0,79 | 0,62 | 0,57 | 46772,39 |
| 47 | Retail trade | 0,45 | 0,36 | 0,29 | 22855,18 |
| 49 | Land & Pipeline transport | 0,00 | 0,00 | 0,00 | 0,00 |
| 50 | Water transport | 0,00 | 0,00 | 0,00 | 0,00 |
| 51 | Air transport | 0,00 | 0,00 | 0,00 | 0,00 |
| 52 | Warehousing | 0,00 | 0,00 | 0,00 | 0,00 |
| 53 | Postal | 0,00 | 0,00 | 0,00 | 0,00 |
| I | Accommodation & Food serv. | 0,93 | 0,86 | 0,73 | 41613,76 |
| 58 | Publishing Act. | 0,00 | 0,00 | 0,00 | 0,00 |
| 59_60 | Media Production | 0,00 | 0,00 | 0,00 | 0,00 |
| 61 | Telecom | 0,00 | 0,00 | 0,00 | 0,00 |
| 62_63 | Computer Programming | 0,00 | 0,00 | 0,00 | 0,00 |
| 64 | Financial Services | 0,00 | 0,00 | 0,00 | 0,00 |
| 65 | Insurance | 0,00 | 0,00 | 0,00 | 0,00 |
| 66 | Auxiliary Financial Serv. | 0,00 | 0,00 | 0,00 | 0,00 |
| L | Real Estate | 1,00 | 1,00 | 1,00 | 207395,10 |
| 69_70 | Legal and Accounting | 0,00 | 0,00 | 0,00 | 0,00 |
| 71 | Architectural and engineering act. | 0,00 | 0,00 | 0,00 | 0,00 |
| 72 | Scientific Research | 0,00 | 0,00 | 0,00 | 0,00 |
| 73 | Advertising and market research | 1,00 | 1,00 | 1,00 | 4237,91 |
| 74_75 | Other professional activities | 0,00 | 0,00 | 0,00 | 0,00 |
| 77 | Rental and leasing activities | 1,00 | 1,00 | 1,00 | 7405,34 |
| 78 | Employment activities | 0,93 | 0,02 | 0,02 | 202,87 |
| 79 | Travel agency & tour operator | 1,00 | 1,00 | 1,00 | 1970,12 |
| 80_82 | Security, landscape, administrative | 0,16 | 0,10 | 0,13 | 4023,71 |
| O | Public administration | 0,00 | 0,00 | 0,00 | 0,00 |
| P | Education | 0,00 | 0,00 | 0,00 | 0,00 |
| 86 | Human health activities | 0,00 | 0,00 | 0,00 | 0,00 |
| 87_88 | Residential care & social work | 0,00 | 0,00 | 0,00 | 0,00 |
| 90_92 | Entertainment, culture, betting | 1,00 | 1,00 | 1,00 | 10276,61 |
| 93 | Sports activities | 1,00 | 1,00 | 1,00 | 7392,37 |
| 94 | Membership organisations | 0,00 | 0,00 | 0,00 | 0,00 |
| 95 | Repair of computers | 0,67 | 0,56 | 0,42 | 769,16 |
| 96 | Other personal services | 0,87 | 0,83 | 0,72 | 14797,54 |
| T | Activities of households as employers | 0,00 | 0,00 | 0,00 | 0,00 |

**Table A.2:** The Italian Prime Minister’s Decree in numbers. Authors elaboration based on the Enterprises economic indicators database provided by the ISTAT. Absolute value added locked is in millions of euro

|  |  | IPMD Lockdonw | | Low-risk scenario | | Medium-risk scenario | | High-risk scenario | |
| --- | --- | --- | --- | --- | --- | --- | --- | --- | --- |
| Codes | Sectors Description | Abs LVA | Rel LVA | Abs LVA | Rel LVA | Abs LVA | Rel LVA | Abs LVA | Rel LVA |
| 1 | Crop and animal production | -6793,42 | -0,23 | -17145,36 | -0,58 | -15003,92 | -0,50 | -4236,52 | -0,14 |
| 2 | Forestry | -691,09 | -0,37 | -1193,33 | -0,63 | -1014,20 | -0,54 | -122,17 | -0,06 |
| 3 | Fishing | -162,56 | -0,16 | -277,69 | -0,27 | -245,85 | -0,24 | -123,16 | -0,12 |
| B | Mining and quarrying | -2230,83 | -0,61 | -3204,18 | -0,88 | -2217,13 | -0,61 | -358,46 | -0,10 |
| 10_12 | Food Product | -5322,84 | -0,19 | -22404,75 | -0,80 | -18094,88 | -0,65 | -3521,27 | -0,13 |
| 13_15 | Textiles | -23321,83 | -0,95 | -19111,83 | -0,78 | -14836,62 | -0,61 | -376,93 | -0,02 |
| 16 | Wood and Cork | -2565,31 | -0,53 | -4346,23 | -0,90 | -3772,36 | -0,78 | -223,20 | -0,05 |
| 17 | Paper Products | -2724,88 | -0,50 | -4768,27 | -0,87 | -3885,32 | -0,71 | -491,67 | -0,09 |
| 18 | Printing and Media | -2733,75 | -0,58 | -4401,67 | -0,93 | -3438,03 | -0,73 | -525,07 | -0,11 |
| 19 | Petroleum Products | -505,09 | -0,20 | -2064,59 | -0,83 | -1606,18 | -0,64 | -154,16 | -0,06 |
| 20 | Chemicals | -6136,07 | -0,51 | -10383,36 | -0,86 | -8784,73 | -0,73 | -537,42 | -0,04 |
| 21 | Pharmaceutical | -675,94 | -0,07 | -6955,07 | -0,75 | -5252,85 | -0,57 | -112,69 | -0,01 |
| 22 | Rubber and Plastic | -10144,33 | -0,81 | -10919,15 | -0,87 | -9116,21 | -0,72 | -689,08 | -0,05 |
| 23 | Other Non-metallic mineral | -9175,85 | -0,93 | -8698,35 | -0,88 | -7422,75 | -0,75 | -614,60 | -0,06 |
| 24 | Basic Metals | -8429,48 | -1,00 | -7434,11 | -0,88 | -6391,63 | -0,76 | -301,17 | -0,04 |
| 25 | Metal products | -28410,97 | -0,95 | -26518,33 | -0,88 | -22646,08 | -0,76 | -1175,25 | -0,04 |
| 26 | Electronics and Computers | -4640,14 | -0,57 | -6362,16 | -0,79 | -4866,53 | -0,60 | -236,82 | -0,03 |
| 27 | Electrical Equipment | -8299,12 | -0,75 | -9093,24 | -0,82 | -7259,58 | -0,65 | -402,38 | -0,04 |
| 28 | Machinery & Equipment | -29927,73 | -0,83 | -28276,94 | -0,78 | -21959,88 | -0,61 | -873,78 | -0,02 |
| 29 | Motor vehicles | -13790,98 | -1,00 | -10649,52 | -0,77 | -8270,79 | -0,60 | -609,85 | -0,04 |
| 30 | Transport equipment | -7400,38 | -1,00 | -5749,13 | -0,78 | -4384,93 | -0,59 | -325,93 | -0,04 |
| 31_32 | Furniture & other manufac. | -9682,56 | -0,71 | -10440,87 | -0,77 | -7979,54 | -0,59 | -260,26 | -0,02 |
| 33 | Installation of machinery | -5309,95 | -0,61 | -7031,25 | -0,81 | -5557,82 | -0,64 | -507,60 | -0,06 |
| D | Electricity & Gas | -9032,58 | -0,38 | -19317,67 | -0,81 | -9856,78 | -0,41 | -2621,10 | -0,11 |
| 36 | Water Collection Activities | -1293,23 | -0,23 | -3791,18 | -0,68 | -1440,28 | -0,26 | -459,95 | -0,08 |
| 37_39 | Waste Collection Activities | -4603,64 | -0,45 | -8132,46 | -0,80 | -4707,03 | -0,46 | -889,85 | -0,09 |
| F | Construction | -43350,20 | -0,66 | -51712,99 | -0,79 | -39817,64 | -0,61 | -2447,19 | -0,04 |
| 45 | Wholesale and retail trade | -9965,58 | -0,59 | -12290,19 | -0,73 | -11855,64 | -0,70 | -9046,89 | -0,53 |
| 46 | Wholesale trade | -57307,90 | -0,70 | -62968,15 | -0,76 | -59955,20 | -0,73 | -44694,46 | -0,54 |
| 47 | Retail trade | -28510,56 | -0,36 | -52078,22 | -0,66 | -51187,86 | -0,65 | -40961,28 | -0,52 |
| 49 | Land & Pipeline transport | -16548,87 | -0,36 | -35672,37 | -0,78 | -18913,43 | -0,42 | -7348,05 | -0,16 |
| 50 | Water transport | -625,57 | -0,17 | -2300,47 | -0,63 | -704,11 | -0,19 | -234,95 | -0,06 |
| 51 | Air transport | -420,59 | -0,24 | -1208,53 | -0,70 | -455,16 | -0,26 | -197,15 | -0,11 |
| 52 | Warehousing | -11869,45 | -0,39 | -24708,29 | -0,81 | -13215,84 | -0,43 | -6012,03 | -0,20 |
| 53 | Postal | -1253,17 | -0,36 | -2778,39 | -0,79 | -1250,76 | -0,35 | -363,90 | -0,10 |
| I | Accommodation & Food serv. | -43724,38 | -0,76 | -57278,09 | -1,00 | -57278,09 | -1,00 | -29421,98 | -0,51 |
| 58 | Publishing Act. | -836,35 | -0,27 | -2035,79 | -0,67 | -703,21 | -0,23 | -259,43 | -0,09 |
| 59_60 | Media Production | -1717,84 | -0,30 | -4149,34 | -0,73 | -1595,33 | -0,28 | -730,32 | -0,13 |
| 61 | Telecom | -4745,09 | -0,26 | -13067,24 | -0,70 | -5008,77 | -0,27 | -1658,98 | -0,09 |
| 62_63 | Computer Programming | -9222,49 | -0,31 | -22163,93 | -0,74 | -9046,94 | -0,30 | -3349,83 | -0,11 |
| 64 | Financial Services | -26695,14 | -0,48 | -46676,97 | -0,83 | -24534,87 | -0,44 | -10421,46 | -0,19 |
| 65 | Insurance | -1816,82 | -0,18 | -6383,19 | -0,63 | -1601,97 | -0,16 | -567,13 | -0,06 |
| 66 | Auxiliary Financial Serv. | -6262,64 | -0,43 | -12811,81 | -0,87 | -6128,10 | -0,42 | -2508,57 | -0,17 |
| L | Real Estate | -207395,10 | -1,00 | -124097,20 | -0,60 | -31460,95 | -0,15 | -16453,96 | -0,08 |
| 69_70 | Legal and Accounting | -25332,89 | -0,53 | -41222,95 | -0,86 | -23955,07 | -0,50 | -7554,47 | -0,16 |
| 71 | Architectural and engineering act. | -8426,48 | -0,49 | -14178,94 | -0,83 | -7990,51 | -0,47 | -2384,39 | -0,14 |
| 72 | Scientific Research | -1361,81 | -0,09 | -8666,83 | -0,56 | -1354,16 | -0,09 | -270,88 | -0,02 |
| 73 | Advertising and market research | -4237,91 | -1,00 | -3839,42 | -0,91 | -2805,82 | -0,66 | -1274,68 | -0,30 |
| 74_75 | Other professional activities | -5899,72 | -0,47 | -10445,92 | -0,83 | -5609,81 | -0,44 | -1849,31 | -0,15 |
| 77 | Rental and leasing activities | -7405,34 | -1,00 | -6270,11 | -0,85 | -3623,45 | -0,49 | -1385,12 | -0,19 |
| 78 | Employment activities | -5284,74 | -0,60 | -7709,94 | -0,87 | -5003,72 | -0,56 | -1909,01 | -0,21 |
| 79 | Travel agency & tour operator | -1970,12 | -1,00 | -1339,78 | -0,68 | -357,39 | -0,18 | -144,53 | -0,07 |
| 80_82 | Security, landscape, administrative | -16548,74 | -0,55 | -24410,08 | -0,82 | -13529,74 | -0,45 | -3596,97 | -0,12 |
| O | Public administration | -6934,89 | -0,07 | -9451,76 | -0,09 | -7156,50 | -0,07 | -2042,68 | -0,02 |
| P | Education | -2317,73 | -0,04 | -3583,49 | -0,06 | -2574,74 | -0,04 | -809,28 | -0,01 |
| 86 | Human health activities | -1454,35 | -0,02 | -1736,31 | -0,02 | -1107,97 | -0,01 | -283,47 | 0,00 |
| 87_88 | Residential care & social work | -1492,47 | -0,11 | -1653,30 | -0,12 | -965,81 | -0,07 | -323,53 | -0,02 |
| 90_92 | Entertainment, culture, betting | -10276,61 | -1,00 | -8337,15 | -0,81 | -6618,24 | -0,64 | -700,25 | -0,07 |
| 93 | Sports activities | -7392,37 | -1,00 | -6166,71 | -0,83 | -4903,57 | -0,66 | -467,33 | -0,06 |
| 94 | Membership organisations | -935,36 | -0,22 | -3352,75 | -0,80 | -2589,07 | -0,61 | -212,36 | -0,05 |
| 95 | Repair of computers | -983,36 | -0,53 | -1446,62 | -0,79 | -1100,55 | -0,60 | -71,55 | -0,04 |
| 96 | Other personal services | -15176,92 | -0,74 | -14863,93 | -0,73 | -10782,07 | -0,53 | -262,65 | -0,01 |
| T | Activities of households as employers | -0,03 | 0,00 | -9220,06 | -0,50 | -0,03 | 0,00 | -0,01 | 0,00 |

**Table A.3:** Absolute (millions of euro) and relative Locked Value Added (LVA) in the 4 scenarios.
